# Beyond lectures: leveraging competition, peer discussion and real-world scenarios in a digital card game to enhance learning of microbiology and immunology concepts

**DOI:** 10.1099/acmi.0.000900.v3

**Published:** 2025-02-13

**Authors:** Michael J. Dillon, James Edwards, Alexandra Hughes, Holly N. Stephenson

**Affiliations:** 1 Peninsula Medical School, University of Plymouth, Drake Circus, Plymouth, PL4 8AA, UK

**Keywords:** digital education, game-based learning, gamification, immunology, infectious diseases, microbiology

## Abstract

Teaching the complex interactions between hosts and pathogens is a fundamental yet educationally challenging aspect of life science and healthcare education. The intricate mechanisms of the immune system can pose significant barriers to students’ understanding of infectious disease diagnosis and treatment. To address this, we used a web-based digital whiteboard platform to design a card-based competitive game called Micro-Immune Battles, aimed at more actively engaging students with microbiology and immunology to better develop their knowledge and underlying concepts. The game facilitates learning through a series of infectious disease scenarios, providing student teams with ‘immune system response cards’ that represent various immune elements. Working in teams, learners must construct sequential card cascades that correctly correspond to the specified pathogen in the scenario. This reinforces the temporal progression of immune responses whilst encouraging the application of theoretical knowledge to practical cases. Scoring is determined by the accuracy and speed of card placements, incentivizing rapid yet correct synthesis of knowledge. Points are deducted for incorrect placements, introducing an element of calculated risk-taking and critical reasoning. Analysis showed statistically significant improvements in microbiology and immunology knowledge after playing the game.

**Impact Statement**

This study introduces a novel educational game designed to enhance medical students’ understanding of microbiology and immunology. The game is structured around infectious disease scenarios and encourages students to apply theoretical knowledge to understand how the immune system fights infection. Our findings show that students enjoyed the game and that their understanding of key immunology concepts improved after playing it. Additionally, they felt more confident in applying this knowledge to real-world scenarios. Students particularly appreciated the gameboard, as it helped them visualize the temporal relationships between various immune components. The broad interest and utility of this game lie in its potential application across various levels of education, from undergraduate students to continuing professional development courses. Though trialled here with medical students, it would be applicable to other groups of learners too. Its use of scaffolding and visualization, combined with an emphasis on critical reasoning and rapid synthesis of knowledge, makes it a versatile tool for diverse educational settings. The significance of this study is twofold: it offers an incremental improvement in educational strategies for microbiology and immunology, and it provides a foundation for future research into game-based learning strategies in medical education.

## Data Summary

Miro, a web-based digital whiteboard application, was used to create this game [1]. Miro is a web-based free software for educators. Supplementary datasets are available from PEARL, an open-access repository (https://doi.org/10.24382/b29cf5d6-3b73-4909-a942-c9793f269667) [2].

## Introduction

The difficulty of effectively teaching the complex interactions between hosts and pathogens is well recognized in medical education literature [3]. Medical students often struggle with the abstract concepts and complex terminology embedded within immunology and microbiology, which can impede their understanding of infectious disease diagnosis and treatment. In response to this educational need, novel teaching methods that incorporate active learning techniques, which promote engagement, critical thinking and the application of knowledge in students, as opposed to passive reception of information enhance cognitive retention and practical skill application have been increasingly advocated [4].

Using games to create engaging, interactive and immersive learning experiences to achieve learning outcomes (LOs) is an emerging approach underpinned by active learning principles. Game-based learning (GBL) strategies encompass a wide range of approaches, including ‘serious games’, where the primary focus is on learning rather than entertainment, and ‘gamification’, which incorporates gaming elements like points and leaderboards to boost motivation and engagement [5]. Digital approaches can improve the design, engagement and scalability of GBL methodologies, especially when working with large class sizes. GBL in medical education can have positive effects on student motivation, engagement, knowledge acquisition and retention [5,6]. Testing in GBL gives students immediate feedback on their performance which allows them to correct errors in understanding. This immediate reinforcement of key principles can lead to improved long-term knowledge retention and increase student satisfaction via positive-feedback mechanisms [7]. Various GBL strategies have been employed to teach medical students immunology and microbiology content, with many focusing on the use of antibiotics [8,9]. To date, few GBL approaches have been employed to teach the complex interplay of host-pathogen interactions.

The aim of this project was to develop a game that addressed the need for innovative teaching tools in the field of infectious diseases. The development of ‘Micro-Immune Battles’, a competitive card-based game, was informed by educational theories that support the use of gamification as a means to increase student engagement and LOs. The project is particularly relevant given the recent introduction of the national Medical Licensing Assessment Applied Knowledge Test, which encompasses core biomedical concepts central to the ‘professional knowledge’ domain. This game-based approach also aligns with Advance HE’s Professional Standards Framework 2023 which calls for the use of diverse teaching methods to cater to different learning styles and to foster a deep understanding of complex subject matter [10].

## Methods

### Participants

Students were recruited from a single academic institution and provided informed consent prior to participation. Students were not required to participate in the entire activity and were free to withdraw consent at any point. Participants were stage 1 undergraduate medical students, who had prior exposure to relevant subject-specific teaching.

### Pre-game assessment

Upon arrival, students completed a 12-question single-best-answer (SBA) multiple-choice questionnaire (Table 1) [2]. This pre-test was designed to assess their baseline knowledge of microbiology and immunology. The questions were designed to reflect real-world scenarios and required the application of theoretical knowledge to practical cases. One hundred thirty-two students consented to sharing their pre-game assessments with the research team.

**Table 1.** Pre- and post-assessment questions, cohort aggregated scores and *z*-scores

Question	Pre-activitypercentage correct responses(*n*=132) (%)	Post-activitypercentagecorrectresponses(*n*=106) (%)	*z*-score
A 28-year-old woman presents to her GP with recurrent abdominal pain, diarrhoea and fatigue. On examination, she is found to have high levels of IgE and eosinophils. What kind of infection does she likely have?	43.9	65.1	3.25 (*P*<0.05)
A 22-year-old male is brought to the emergency department with a large, painful swelling on his arm after a camping trip. The culture of the wound grows *Staphylococcus aureus*. In the context of bacterial infections, what is the primary function of neutrophils?	70.5	87.7	3.21 (*P*<0.05)
During a lecture, a medical student asks about the role of Toll-like receptors (TLRs) in the immune response. Which of the following best describes the function of TLRs?	56.1	83.0	4.43 (*P*<0.05)
A 40-year-old woman is diagnosed with a severe fungal infection. Which of the following cells is particularly important in forming the initial innate immune response to fungal pathogens?	30.3	49.1	2.95 (*P*<0.05)
A 30-year-old patient presents with a history of frequent viral infections. On investigation, it is found that their NK cells have reduced activity. Which function of NK cells is critical in controlling viral infections?	56.8	67.9	1.75 (*P*<0.05)
A researcher is studying the immune response to a novel virus and observes that CD8+ T cells play a significant role in the clearance of infected cells. Which of the following best describes the primary function of CD8+ T cells in viral infections?	68.9	77.4	1.45 (*P*>0.05)
A 10-year-old boy presents with a history of frequent infections and poor wound healing. Upon further examination, he is diagnosed with a genetic disorder affecting his complement system. Which one of the functions of the complement system is important for enhancing protection against pathogens?	73.5	74.5	0.182 (*P*>0.05)
A patient diagnosed with chronic lymphocytic leukaemia shows an increased number of defective B cells. Which of the following functions would be most compromised in this patient?	20.5	38.7	3.10 (*P*<0.05)
During an immunology class, a student asks about the function of dendritic cells. Which of the following best describes the primary role of dendritic cells in the immune response?	61.4	74.5	2.15 (*P*<0.05)
A 45-year-old man with a history of recurrent nasal infections is found to have elevated levels of a particular antibody isotype in his serum. Which antibody isotype is typically found in mucosal tracts?	35.6	58.5	3.52 (*P*<0.05)
A patient diagnosed with HIV is found to have a severely diminished CD4+ T cell count. Which aspect of the adaptive immune response is most directly affected by the depletion of CD4+ T cells?	31.8	56.6	3.84 (*P*<0.05)
A 58-year-old woman with diabetes presents with a non-healing ulcer on her foot. A biopsy of the ulcer shows macrophages infiltrating the tissue. What is the primary role of macrophages in wound healing?	71.2	74.5	0.571 (*P*>0.05)
Overall assessment performance	51.7	67.3	2.43 (*P*<0.01)

### The educational game

**Fig. 1.**
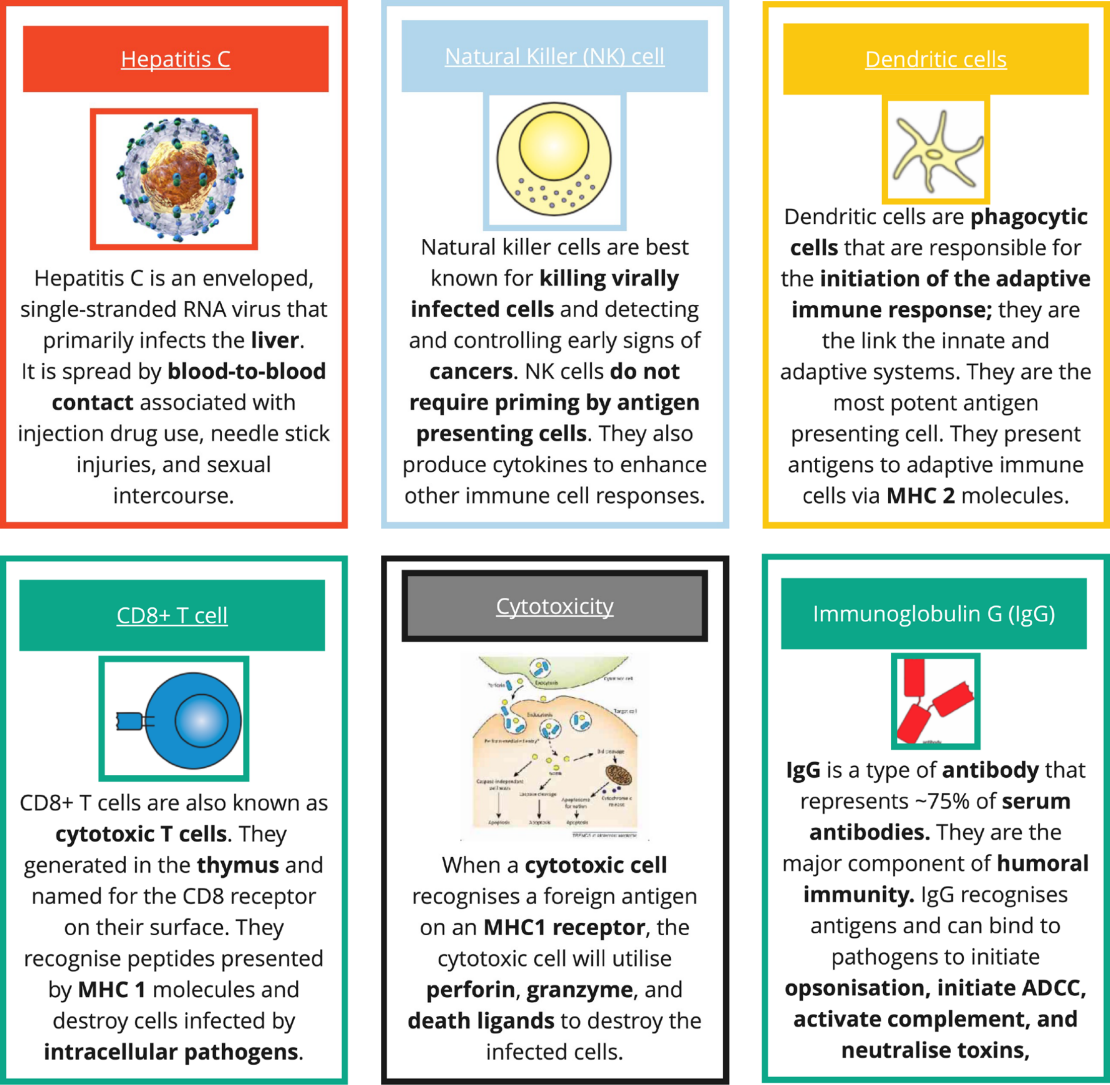
Exemplar game cards. These cards represented various elements of the scenario, such as an infectious disease, various immune cells and how these are all related.

Following the pre-test, students were introduced to the educational game, which structured learning around a series of infectious disease scenarios. Students were divided into teams and provided with ‘immune system response cards’. These cards represented various elements of the immune system, such as macrophages, T cells and antibodies (Fig. 1).

**Fig. 2.**
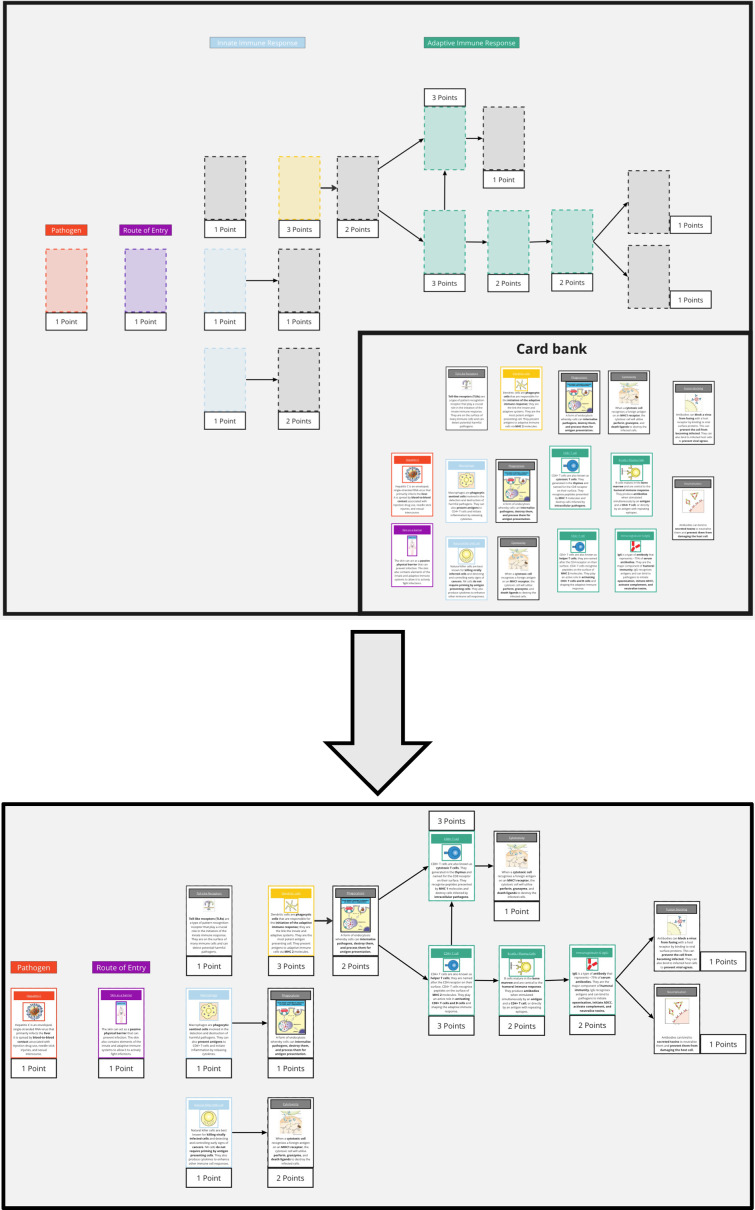
Exemplar initial game scenario. Participants are tasked with arranging in a way that demonstrates how the immune system interacts with infectious diseases. The initial scenarios are simply designed and use colour-coded cards to provide a visual cue that guides participants into correct card placement. As the game progresses, scenarios become more complicated and interconnected, with distractor cards added and colour coding removed.

Teams were tasked with constructing sequential card cascades that accurately corresponded to the pathogen specified in each scenario (Fig. 2). This process was intended to reinforce the temporal progression of immune responses and encourage the application of theoretical knowledge to practical situations.

An incremental scaffolding approach was used; the initial scenarios presented were straightforward and simple, utilizing colour-coded cards to provide a visual cue and guide decision-making (Fig. 2). Later scenarios became more intricate, with more interconnected pieces. The colour-coding was gradually removed, and distractor cards were added to the bank, requiring students to apply previous learning to discern which immune components were applicable to the current infection scenario. Teams were given approximately 10 min for each scenario, and all teams were able to complete each scenario within the time limits. When a team completed their scenario, they then explained their findings to an academic lead who discussed the results with them and scored their work. Three bonus points were awarded to the team that completed the scenario most accurately. When multiple teams achieved the same level of accuracy, the bonus points were awarded to the team that completed the scenario first.

### Game mechanics

Scoring in the game was determined by both the accuracy and speed of card placements. Teams received points for correct placements and deductions for incorrect ones, thereby introducing elements of calculated risk-taking and critical reasoning. This dual emphasis on speed and accuracy was designed to incentivize rapid yet correct synthesis of knowledge.

The score assigned to each card placement was decided by the development team based on intuition and experience. The goal was to make certain placements more valuable and thus drive engagement with concepts that students have traditionally struggled with.

### Post-game assessment

After completing the game, students took the same 12-question SBA-style multiple-choice questionnaire (Table 1) [2]. This post-test aimed to measure any changes in students’ knowledge and understanding as a result of participating in the game. One hundred six students consented to share their post-game assessments with the research team.

### Data analysis

The primary outcome measure was the difference in pre- and post-test scores, which was analysed to determine the effectiveness of the game as a pedagogical tool. Statistical analyses were conducted using cohort-aggregated scores with a proportional *z*-test to evaluate the significance of any observed changes in scores (Table 1).

### Additional information

Within the wider curriculum, this session was used as a final consolidation within a 2-week teaching block on infection and immunity. Content experts were present to pose questions, assure quality and facilitate discussion during the review rounds. This session was not meant to teach new concepts, but rather to reinforce previously introduced ideas. The involvement of multiple content experts supported the integration of knowledge during the sessions.

### Limitations

Cohort-aggregated pre- and post-test scores mean we are unable to match individual responses across the two time points, thus making paired statistical analysis impossible. This means that our analysis does not account for within-individual variance, rather it acts as a proxy measure of change across time points for the entire cohort.

All participants were treated the same in this study. There were no control groups or alternative test versions. Therefore, it is difficult to account for any improvements purely due to the repetition of the test.

The study’s reliance on a single institution may limit the generalizability of the findings. Additionally, the short-term nature of the intervention precludes any conclusions about long-term retention of knowledge.

## Results

### Participants’ learning

A proportional *z*-test analysis showed statistically significant improvements in the percentage of correct answers for 9 out of 12 questions (*P*<0.05) (Table 1). The overall assessment performance also showed a statistically significant improvement, with the average percentage of correct answers increasing from 51.7% in the pre-activity questionnaire to 67.3% in the post-activity questionnaire (*P*<0.01) (Table 1).

### Participation enjoyment

The game was well-received, with 52% (*n*=53), regarding the game as either ‘Good’ or ‘Very good’. Similarly, 54% (*n*=54) of students felt that the game enhanced their comprehension of microbiology and immunology concepts, and 43% (*n*=44) expressed confidence in their ability to apply these concepts to real-life scenarios. The distribution of responses across the Likert scale is summarized in Table 2.

**Table 2.** Post-activity feedback questionnaire response distribution

Question	Scale/no. of responses				
	**Very poor**	**Poor**	**Neutral**	**Good**	**Very good**
How did you find the overall experience of participating in the ‘Micro-Immune Battles’ game?	6	17	27	32	21
	**Definitelyno**	**No**	**Unsure**	**Yes**	**Definitelyyes**
Do you feel that the game helped you understand immunology and microbiology concepts better?	5	19	25	24	30
	**Very unconfident**	**Unconfident**	**Unsure**	**Confident**	**Very confident**
How confident do you feel about applying the concepts you learnt from the game to real-world contexts?	15	19	25	31	13

### Qualitative feedback

Qualitative comments (*n*=54) offered insights into specific features of the game that were particularly effective or ineffective. For example, several students (*n*=18) specifically mentioned that the flow chart layout was helpful for improving their understanding.

*The map felt like it stuck in my brain well*. [Participant 1]

*I loved being able to visualise and fill in the pathway. It was brill*. [Participant 2]

*[It was] great to be able to put our learning to good use and see the flow chart of how the immune response works*. [Participant 3]

Similarly, multiple students (*n*=6) appreciated having staff available on to score their answers and discuss the scenarios with them.

*Teachers walking around was helpful to clarify*. [Participant 4]

*Best part was staff walking round and explaining things. This really helped*. [Participant 5]

Amongst those who chose the neutral or negative options, *n*=15 would have preferred using a physical, card-based game instead of the digital Miro board. These comments suggested that it was difficult to work collaboratively on small screens.

*Don’t use [Miro] board, use paper instead as it is easier to sort out and work with multiple people*. [Participant 6]

*Hard to work on a digital platform - felt uncoordinated*. [Participant 7]

*It was fun, although doing it digitally makes it difficult to read and see what other people are doing specifically*. [Participant 8]

Next, some (*n*=14) felt that the team sizes were too large and felt that they would have benefitted from small groups.

*Sharing the resource between 9 [team members] was quite difficult*. [Participant 9]

*I liked the session but think it didn’t work so well in such a big group*. [Participant 10]

*I think the concept was good but the writing was small and in smaller groups could have worked better*. [Participant 11]

Next, *n*=3 participants felt that there was too much scaffolding, making the game too easy. One participant suggested that the timing of the session kept them from engaging as they were preoccupied with studying for their mid-term exams.

*Less scaffolding, e.g. I’m not very good at immunology but I could logically work out the answers so it felt a bit useless* [Participant 12]

*…just end up matching colours…* [Participant 13]

*I also liked the colour matching but it meant we didn’t really read the cards as much*. [Participant 11]

Finally, there were 34 responses exploring post-session participant confidence in applying the learning to real-world scenarios. Interestingly, 33% (*n*=11) felt that they needed to spend more time independently studying before they could feel confident.

*I feel I still need to independently consolidate my learning on this topic*. [Participant 14]

Still need to do a bit of independent learning. [Participant 15]

*Need more time to go over the content*. [Participant 15]

## Discussion

Educating medical students about microbiology and immunology is a well-recognized challenge [11], both in terms of enhancing enjoyability, developing literacy [12] and developing links through to clinical relevance [3]. The objective of this project was to address these challenges by creating an educational resource designed to develop specific *knowledge* in relation to the immune system and develop an *understanding* of how the different elements of the immune system work together when presented with different pathogens. The GBL, rule-based style, provides an opportunity for knowledge consolidation, and the activity mimics some of the ‘rules’ that the immune system follows when fighting infection. This framework helps learners see how the ‘rules’ fit together, somewhat akin to concept mapping, which facilitates the visualization of relationships between concepts and enhances comprehension and retention [13]. Similar activities and resources have been developed previously to supplement and reinforce more traditional didactic teaching environments and have also met with success [14].

A goal within medical education (and more broadly within life science education) is the integration of knowledge. Traditional teaching environments with one topic and one content expert are not always well equipped to achieve this aim. Knowledge integration can be both horizontal across disciplines, for example, integrating knowledge between discrete scientific disciplines or vertical, for example, integrating ‘basic’ science knowledge into clinical practice [15]. Activities, such as those presented here, can help achieve integration by allowing students to draw on their knowledge and understanding of microbiological, cellular, immunological, molecular and biochemical concepts – many of which may have been taught in discrete settings – and bring them together to apply their learning. The setting of the human body and the clinically orientated formative assessment questions provided clinical relevance and integration to learners.

Our results show that the game helped students develop and consolidate specific knowledge as assessed through answering SBA questions. Questions were written by subject experts as an opportunity to formatively assess students on topics covered in the prior 2 weeks. The overall trend in performance saw improvements in 9 out of 12 questions asked. Of those questions that saw little improvement in their performance, these had a pass rate of >69% in the pre-activity assessment. Looking at the performance of specific questions, topics such as the function of specific immune cells (question 2, neutrophils, question 6, CD8+) and complement (question 7) performed well. This is arguably unsurprising as this knowledge would have been frequently addressed in specific teaching, preparatory sessions and LOs linked to formal teaching sessions over the preceding 2 weeks. Some of the poorer-performing questions (question 4, response to fungi, question 8, leukaemia) were arguably more ‘stretch’ questions which, though touched on topics introduced in the preceding 2 weeks, are not taught extensively and are explored further in stage 2 of the programme. Nevertheless, these questions provide an important place to introduce some new ideas and can help students see areas to address in their independent study [16].

Not only did students improve their specific knowledge, but we can also see that students largely found the experience positive and felt that they understood microbiology and immunology better and that their confidence in this understanding had improved. Confidence plays a crucial role in the learning process. It affects a student’s willingness to engage with challenging material and their persistence in overcoming difficulties [17]. When students feel confident, they are more likely to participate actively, ask questions and take intellectual risks that are essential for deep learning.

That said, many of our participants still felt that they needed to revise independently before they could declare themselves fully confident. Independent study is crucial as it allows learners to consolidate knowledge and develop self-directed learning skills, which are essential for lifelong learning. The game was initially designed using the digital whiteboard platform Miro to facilitate post-session access for students. However, this approach may have hindered in-session engagement, as the integration of technology in educational settings can sometimes complicate interaction and impede active learning if not carefully designed and implemented [18]. Additionally, the game was conducted in teams of nine, a group size that some students felt was too large for effective learning. Research indicates that smaller groups, typically comprising three to four members, are more conducive to collaborative learning, as they encourage active participation and reduce the likelihood of social loafing [19]. Larger groups can lead to decreased individual accountability and diminished engagement, thereby hindering the collaborative process. In future implementations, we plan to provide a physical version of the game for in-person sessions and a digital version for independent study, whilst limiting group sizes to approximately four to six members to enhance collaborative efficacy.

### Future work

Going forward, it would be beneficial to explore the ‘what’ further – i.e. what about the session was useful? Were there specific tasks that occurred that were more useful than others? Were there new ideas introduced in the process of completing these tasks that might have been new to learners in the room? Additionally, the design of the activity required students to work in groups of around eight peers to work through the tasks. Peer-to-peer learning provides an opportunity to share uncertainties and an informal avenue for teaching to occur amongst the group; it is a powerful tool for students to develop and consolidate their understanding of difficult topics in a low-stress environment [20]. Exploring these ideas may require more targeted and specific questions included in the post-activity feedback questionnaire.

## Conclusion

Micro-Immune Battles represents an advancement in microbiology and immunology pedagogy. This game benefits from a structured approach, leveraging visualization, scaffolding, GBL and competition into an activity that can enhance LOs across diverse groups of learners. It effectively bridges the gap between theoretical knowledge and practical application, addressing the pedagogical challenges inherent in teaching complex host-pathogen interactions. Our study demonstrates that the game not only leads to measurable improvements in learner understanding of key immunological concepts but also enhances their enjoyment and engagement of the topics.

## Abbreviations

GBL: game-based learning
LO: learning outcome
SBA: single best answer

## References

[1] Miro (2024). Miro. https://miro.com.

[2] Dillon MJ, Edwards J, Hughes A, Stephenson HN (2024). Beyond lectures micro-immune battles evaluation. Project Dataset.

[3] Haidaris CG, Frelinger JG (2019). Inoculating a new generation: immunology in medical education. Front Immunol.

[4] Freeman S, Eddy SL, McDonough M, Smith MK, Okoroafor N (2014). Active learning increases student performance in science, engineering, and mathematics. Proc Natl Acad Sci U S A.

[5] Xu M, Luo Y, Zhang Y, Xia R, Qian H (2023). Game-based learning in medical education. Front Public Health.

[6] Gentry SV, Gauthier A, L’Estrade Ehrstrom B, Wortley D, Lilienthal A (2019). Serious gaming and gamification education in health professions: systematic review. J Med Internet Res.

[7] Putz L-M, Hofbauer F, Treiblmaier H (2020). Can gamification help to improve education? Findings from a longitudinal study. *Computers in Human Behavior*.

[8] Tsopra R, Courtine M, Sedki K, Eap D, Cabal M (2020). AntibioGame®: a serious game for teaching medical students about antibiotic use. J Med Inf.

[9] Imwattana K, Methawirune A, Voralakkulchai I, Ngamskulrungroj P (2023). BactoBattle: a game-based learning companion for medical bacteriology. *Access Microbiol*.

[10] Advance HE (2023). Professional standards framework for teaching and supporting learning in higher education 2023. https://advance-he.ac.uk/knowledge-hub/professional-standards-framework-teaching-and-supporting-learning-higher-education-0.

[11] Manglik N, Dudrey EF, Baatar D, Piskurich JF (2017). Immune response to bacteria: an integrated learning module to enhance preclinical students’ competency in immunology. MedEdPORTAL.

[12] Siani M, Dubovi I, Borushko A, Haskel-Ittah M (2024). Teaching immunology in the 21st century: a scoping review of emerging challenges and strategies. Int J Sci Educ.

[13] Sannathimmappa MB, Nambiar V, Aravindakshan R (2022). Concept maps in immunology: a metacognitive tool to promote collaborative and meaningful learning among undergraduate medical students. J Adv Med Educ Prof.

[14] Su T, Cheng M-T, Lin S-H (2014). Investigating the effectiveness of an educational card game for learning how human immunology is regulated. *CBE Life Sci Educ*.

[15] Wijnen-Meijer M, van den Broek S, Koens F, Ten Cate O (2020). Vertical integration in medical education: the broader perspective. BMC Med Educ.

[16] Harland T (2003). Vygotsky’s zone of proximal development and problem-based learning: linking a theoretical concept with practice through action research. Teach High Educ.

[17] Robbins SB, Lauver K, Le H, Davis D, Langley R (2004). Do psychosocial and study skill factors predict college outcomes? A meta-analysis. Psychol Bull.

[18] Godsk M, Møller KL (2024). Engaging students in higher education with educational technology. *Educ Inf Technol*.

[19] Corrégé J-B, Michinov N (2021). Group Size and Peer Learning: Peer Discussions in Different Group Size Influence Learning in a Biology Exercise Performed on a Tablet With Stylus. Front Educ.

[20] Ravanipour M, Bahreini M, Ravanipour M (2015). Exploring nursing students’ experience of peer learning in clinical practice. J Educ Health Promot.

[21] Microbiology Society (2024). Annual Conference 2024 Poster Abstract Book. https://microbiologysociety.org/static/e2a41dbb-eafd-4056-89611c63c97bd6c7/AC24Poster-abstracts-booklet.pdf.

